# Effect of *Lycium Barbarum* (Wolfberry) *Polysaccharides* on Preserving Retinal Function after Partial Optic Nerve Transection

**DOI:** 10.1371/journal.pone.0081339

**Published:** 2013-12-10

**Authors:** Patrick H. W. Chu, Hong-Ying Li, Man-Pan Chin, Kwok-fai So, Henry H. L. Chan

**Affiliations:** 1 Laboratory of Experimental Optometry (Neuroscience), School of Optometry, The Hong Kong Polytechnic University, Hong Kong SAR, China; 2 Department of Anatomy, Research Centre of Heart, Brain, Hormone and Healthy Aging, The State Key Laboratory of Brain and Cognitive Sciences, The University of Hong Kong, Hong Kong SAR, China; 3 Institute of Active Ageing, Faculty of Health and Social Sciences, The Hong Kong Polytechnic University, Hong Kong SAR, China; The University of Western Australia, Australia

## Abstract

*Lycium Barbarum Polysaccharides* (LBP) are the active components of Wolfberry (a traditional Chinese medicine) which has long been used for improving visual function. This study aims to investigate localized changes of retinal function in a partial optic nerve transection (PONT) model, and effects of LBP on visual function. The multifocal electroretinograms (mfERG) were obtained from 30 eyes of 30 Sprague-Dawley rats. The rats were divided into 6 groups (five treatment groups and one control group). Starting from the first day of the experiment, the rats in the (PONT+LBP) group and the (LBP) group were dosed with LBP; rats in the (PONT+PBS (phosphate buffered saline)) group and the (PBS) group were dosed with PBS via nasogastric tube every day until euthanized. The dorsal part of the optic nerve was transected in the (PONT), (PONT+LBP) and (PONT+PBS) groups at the end of week 1 (day 7 after LBP or PBS feeding began). The mfERG was measured at three time points: week 2, week 3 and week 5. Significant reduction of P1 and PhNR amplitudes of the mfERG were observed in all retinal regions a week after PONT. Feeding with LBP prior to PONT preserved retinal function. All mfERG responses returned to the normal range in the superior retina, which corresponds to the transected dorsal region of the optic nerve, while most of the inferior retinal responses were significantly increased at week 4 after PONT. The ventral part of the retina had secondary degeneration which was not only limited to the ganglion cell layer, but is a widespread effect affecting the outer retina. LBP altered the functional reduction caused by PONT by regulating the signal from the outer retina.

## Introduction

Glaucoma treatment will always be challenging as the retinal ganglion cells lost due to glaucomatous damage cannot be recovered. An important treatment goal is to prevent further damage to retinal ganglion cells after diagnosis of glaucoma.

Neuroprotection is a current therapeutic strategy that prevents retinal ganglion cells from undergoing glaucomatous degeneration. Lowering intraocular pressure (IOP) is the most common therapy for glaucoma and is effective in reducing the progression of glaucoma, [1] but it has been reported that it is not possible to totally halt the progression of glaucomatous optic neuropathy. About 45% of patients still suffer glaucomatous degeneration 6 years after diagnosis and treatment, even when IOP has been well-controlled. [1] Therefore, considerable efforts have been made to develop neuroprotective agents to slow any degenerative process(es) in glaucoma. *Fructus Lycii*, the dried mature fruit of *Lycium Barbarum Linn*, the Wolfberry, is a traditional Chinese medicine which has long been used for improving visual function. [2] *Lycium barbarum polysaccharides* (LBP), the active component of *Fructus Lycii*, has been reported to have a neuroprotective effect in reducing cortical neuronal death in Alzheimer’s disease [3], in preventing retinal ganglion cell loss in ocular hypertension [4], [5], [6] and in retinal ischemia/reperfusion injury. [7] This herb is believed to be a potential candidate for the prevention of neurological disorders.

It has been suggested that secondary degeneration of retinal ganglion cells, degeneration beyond the site of primary insult, plays an important role in the progression of glaucomatous damage, as the apoptosis of retinal ganglion cells can still progress even after elevated IOP has declined in an ocular hypertension model. [8] However, an ocular hypertension model is not ideal for investigating secondary degeneration as the whole retina suffers from primary injury caused by the elevated IOP. A partial optic nerve transection (PONT) model has been developed for studying secondary degeneration of retinal ganglion cells. [9], [10] In this model, only part of the dorsal optic nerve is cut; the PONT model allows good separation of secondary degeneration from the directly injured retinal ganglion cells. Since the ganglion cells in the dorsal retina project their nerve axons mainly along the dorsal optic nerve in rodents [10] and primates, [9] most of the ganglion cells lost in superior retina after PONT are mainly caused by direct injury and secondary degeneration is mainly found in the inferior retina. [11], [12], [13] This model provides a feasible platform for investigating the mechanism [12], [13] and pharmacologic interventions [13], [14], [15], [16] of secondary degeneration in glaucoma.

Although the neuroprotective effect of LBP on the degeneration of retinal ganglion cells in ocular hypertension [4], [5], [6] or the secondary degeneration of retinal ganglion cells after PONT [11] has been shown histologically, its effect on preserving visual function is still uncertain. In this study, the multifocal electroretinogram (mfERG) which can record information both from inner retina (photopic negative response) [17] and outer retina (N1 and P1), [18], [19] was applied to measure the localized changes of retinal function in a PONT model and the effect of intervention with LBP was assessed. The mfERG allows for recording multiple local retinal responses within a short time period, [20] and it is widely used in glaucoma investigation in both human [21], [22], [23], [24] and animal studies. [25], [26] Therefore, it is suitable for evaluating the changes of retinal function due to localized degeneration caused by PONT and any treatment effects of LBP. This paper is a companion paper to that of Li et al. 2013 [11] which provides histological data for the same experiment.

## Materials and Methods

### Animals

The mfERG recordings were obtained from 30 eyes of 30 twelve-week-old (250–280 g) Sprague-Dawley (SD) rats (Laboratory Animal Unit, The University of Hong Kong, Hong Kong). The rats were equally divided into 6 groups (Table 1) and only the right eyes were used in this study. All the rats were reared in a temperature-controlled room on a 12-hr light/12-hr dark cycle, with food and water supplied *ad libitum*, during the 5 weeks of this study. All the rats in (PONT+LBP), (LBP) and (PONT+PBS), (PBS) groups were administered LBP (1 mg/kg) or phosphate buffered saline (PBS) respectively, via a nasogastric tube every day until euthanization (Table 1). The dosage of LBP followed the preparation and the procedures described by Yu *et al*. in 2007. [27] The PONT surgery was performed in the (PONT), (PONT+LBP) and (PONT+PBS) groups at the end of week 1 (day 7 after start of LBP or PBS feeding). The mfERG was measured for all 6 groups at three time points: week 2 (day 14), week 3 (day 21) and week 5 (day 35). All experimental and animal care procedures adhered to the ARVO Statement for the Use of Animals in Ophthalmic and Vision Research and were approved by the Committee on the Use of Live Animals in Teaching and Research of The University of Hong Kong (CULATR #1850-09 and #1996-09) and the Animal Ethics-subcommittee of The Hong Kong Polytechnic University (ASESC No. 09/15).

**Table 1:** Experimental grouping for different treatments.

Group	Treatment	Objectives	No. of rats
Control	No treatment	Control	5
PONT	PONT only	Effect of PONT on mfERG	5
PONT+LBP	PONT with LBP	Effect of LBP on PONT	5
PONT+PBS	PONT with PBS	Effect of PBS on PONT	5
LBP	LBP only	LBP control	5
PBS	PBS only	PBS control	5

PONT – partial optic nerve transaction; LBP – *Lycium Barbarum Polysaccharides*; PBS – phosphate buffered saline.

### PONT Surgery

**Figure 1:**
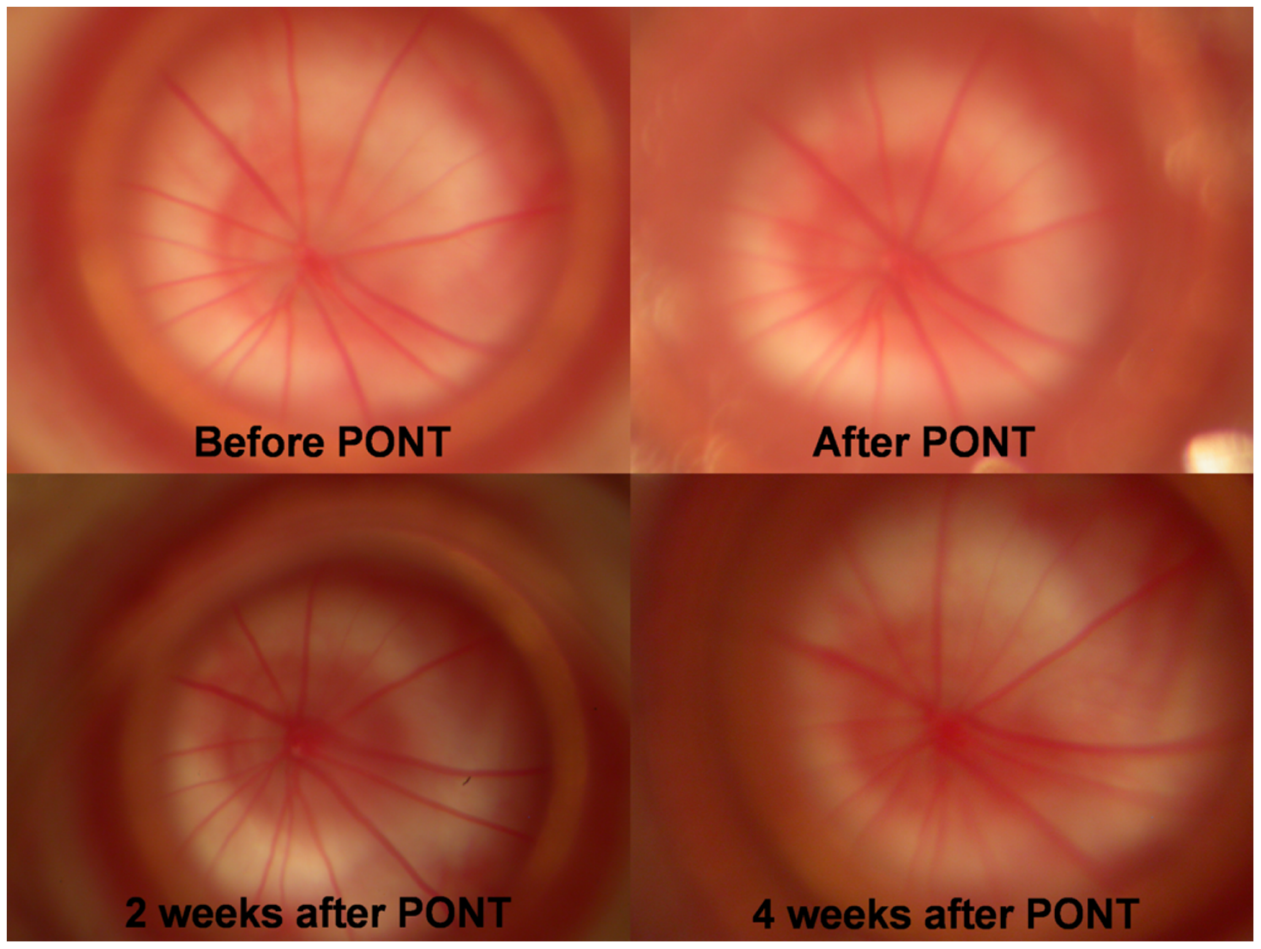
Fundus photographs. The photos of the fundus from the right eye of a SD rat before and after PONT.

The PONT procedure was the same as that described by Li et al. [11] Rats were anesthetized with intraperitoneal injection of ketamine (80 mg/kg) and xylazine (8 mg/kg); 0.5% proparacaine hydrochloride was used for topical anesthesia before the surgery. The upper eye lid was raised and an incision was made in the superior conjunctiva. The eye was gently retracted with forceps, exposing the optic nerve. The dural sheath of the optic nerve was opened and the dorsal part of the optic nerve was transected (200 µm depth of cut marked by a pair of Spring Vannas scissors (15000-08, F.S.T., Heidelberg, Germany)) about 1 mm behind the eyeball using a diamond radial keratotomy knife (G-31480, Geuder AG, Hertzstrasse, Heldelberg, Germany). After the procedure, ophthalmic ointment (0.3% tobramycin) was applied to the cornea to prevent inflammation, and the fundus was inspected to ensure that the retinal blood supply was not compromised by the surgery (Figure 1).

### Multifocal ERG (mfERG) Stimulation

**Figure 2:**
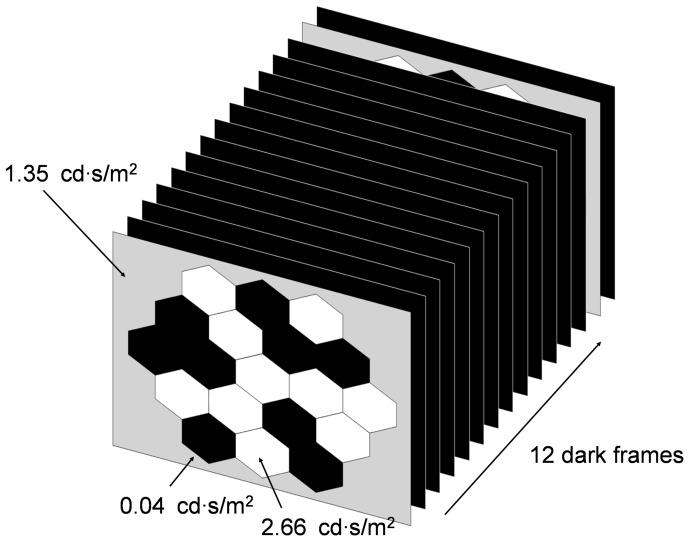
Schematic diagrams of slow mfERG stimulation paradigm. The stimulation paradigm consists of 12(2^12^-1) times in the whole measuring period at weeks 1, 2 and 4.

A 19-hexagon non-scaled stimulus pattern, driven by the VERIS software (ver. 5.01) from Electro-Diagnostic-Imaging (San Mateo, California, USA), was presented on a 22-inch liquid crystal display monitor (model: vx2260wm; ViewSonic, USA). The working distance from the screen to the tested eye was 15 cm, so the stimulus pattern subtended a visual angle (both horizontal and vertical) of about 80°. The mfERG stimulation followed a slow stimulation paradigm (Figure 2), where the stimulation sequence contained 13 video frames (each frame lasted 13.3 ms with a frame rate of 75 Hz). During the first video frame with multifocal flashes, each hexagon was either flashed (2.66 cd·s/m^2^) or dark (0.04 cd·s/m^2^) according to the selected pseudo-random binary m-sequence (2^12^-1 frames in length), and this multifocal flash frame was followed by 12 dark video frames before the next cycle of stimulation. This slow stimulation paradigm reduced the flash rate to approximately 6 Hz which provides enough time for the recovery of the photoreceptors in rats. [28], [29] The average luminance of the multifocal flash frame was about 1.35 cd·s/m^2^ and the background was set to this value. The recording time was approximately 12 min.

### Recordings

Before testing, the animal was kept in darkness overnight, the pupils of the tested eyes were fully dilated with 1% tropicamide, and the ocular surface was anaesthetized with 0.4% benoxinate HCl. The eyelids of the tested eye were held by an eye speculum. A monopolar contact lens electrode (Mayo, Inazawa, Japan) was used as the active electrode. It was placed on the cornea with ocular lubricant and Grass subdermal F-E7 electrodes (Astro-Med, West Warwick, Rhode Island, USA) were applied subcutaneously at the tail and at the temporal canthus of the tested eye as ground and reference electrodes, respectively. The rats were anesthetized using 2% isoflurane with 98% oxygen supply through a mask throughout the measurement. The refractive error of the tested eye after placing the contact lens electrode was fully corrected with trial lenses for the viewing distance (around −2 to −4 DS). Before recording, the central hexagon of the stimulation pattern was aligned with the optical axis of the tested eye; this allows approximate location of the optic nerve head response of the tested eye. The mfERG signals were amplified using a Grass amplifier (model CP122 bench-top style amplifier; Grass Instruments, Quincy, Massachusetts, USA) with band pass 1–300 Hz and gain x20,000. After the mfERG recording, the rat was allowed to recover from anesthesia before being placed back in its cage.

### Intravitreal Injections

Pharmacological blockage of inner retinal activity was performed for the control group of rats at week 5 after recording for the experiment proper. Intravitreal injections (2 µL), with a sterile 30-gauge needle attached to a 25 µL Hamilton micro-syringe (Hamilton Company, Reno, NV, USA), were made 1 mm posterior to the superior limbus through the sclera at an angle of 45° to avoid contact with the crystalline lens. Assuming that the vitreal volume is 40 µL, the intravitreal concentrations of the pharmacologic agents (Sigma-Aldrich Co., St Louis, MO, USA) used were: Tetrodotoxin (TTX: 5 µM) and N-methyl-D-asparatic acid (NMDA: 4 mM). These concentrations are sufficient to have the desired effects on the flash ERG or mfERG in rats. [30], [31] All animals were anesthetized during each intravitreal injection and then allowed recovery before the mfERG recording in order to minimize the effect of prolonged anesthesia. The mfERG recordings were conducted at least one hour after each drug administration to allow stabilization of the drug effect.

### Data Analysis

**Figure 3:**
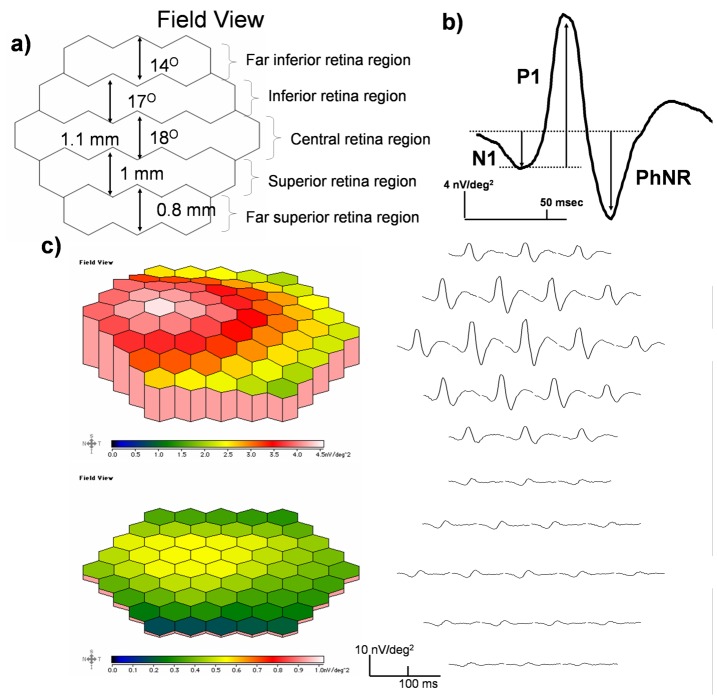
Diagrams of mfERG waveform, traces array and three-dimensional plot. **a)** The 19 hexagons were grouped into 5 regions where the central region subtended about 18° in field size and was representing the central 1.1 mm retinal response; the whole pattern covered around 5.8 mm in diameter of the rat retina. [38] **b)** A typical averaged response from the visual streak of a SD rat eye and the measurement of the response amplitudes is illustrated. **c)** Three-dimensional field view topography and response traces from the right eye of a SD rat in the control group (top panel); the three-dimensional field view topography and response traces from the right eye of a SD rat in the PONT group 4 weeks after PONT are shown at the lower panel.

First-order kernel mfERG responses were analyzed using the VERIS software. The 19 individual mfERG responses without spatial averaging from each rat eye were grouped into 5 regions, representing the superior and inferior retinal function (Figure 3a). The mfERG findings are presented as peak-to-peak response amplitude measurements of the relevant components (N1, P1 and PhNR: see Figure 3b) in the first-order kernel analysis. Implicit times are not shown in this study because there were no significant variations in implicit times between conditions. Comparisons of mfERG responses under various conditions were made using ANOVA with Bonferroni *post hoc* test. The effect of PONT on mfERG was examined by comparing the control and the PONT group, where the LBP control and the PBS control group were used to assess the functional effect of LBP or PBS on PONT respectively.

## Results

The typical first-order kernel mfERG response waveforms from the SD rat are shown in Figure 3b. As with the primate mfERG response, the waveform contains a trough (N1) at around 25 ms, followed by a major positive component (P1) at around 55 ms, and a photopic negative response (PhNR) which can be observed at around 75 ms. The topographical mfERG response demonstrated a stronger retinal function along the visual streak with a peak in the nasal field in both conditions with and without PONT (Figure 3c). In addition, there was no change in implicit time of the mfERG response in any condition.

### Effect of PONT

**Figure 4:**
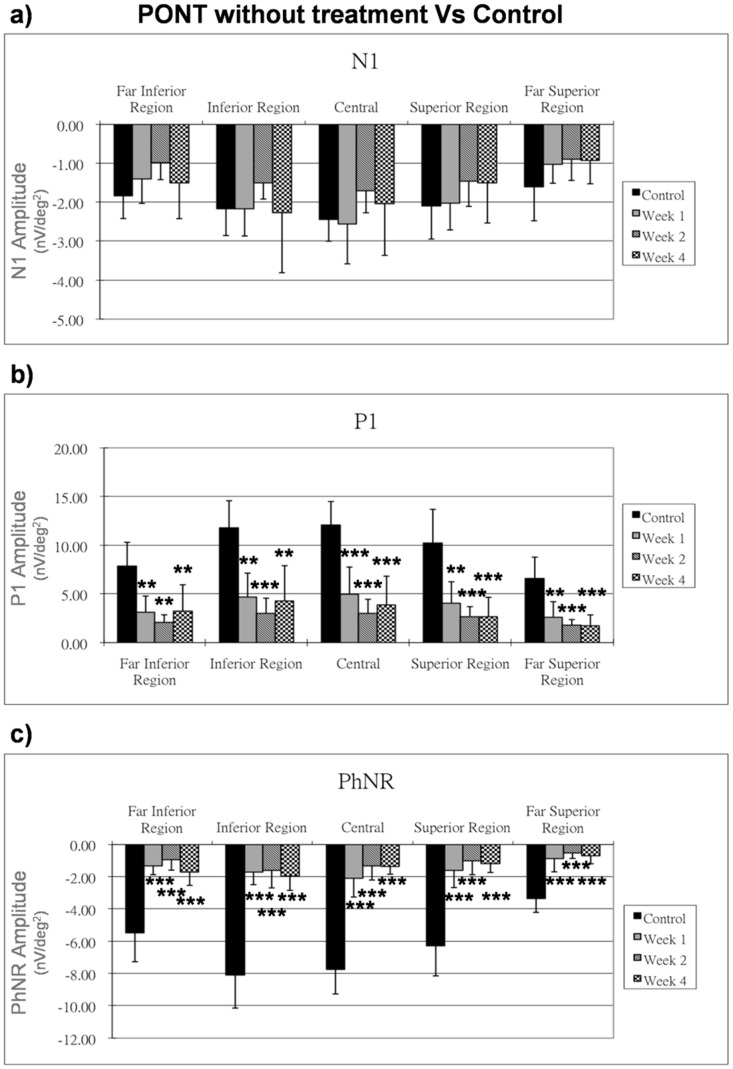
Effect of PONT on mfERG. The mfERG responses (nV/deg^2^) at one, two and four weeks after PONT are shown. The responses are compared with those of the control group. Bars = 1 SD; *p<0.05; **p<0.01; ***p<0.001.

In the control group, the amplitude of all mfERG components (including N1, P1 and PhNR) among three time points of measurement were compared and no significant differences were noticed. All the mfERG responses from the other treatment groups have been compared with the first measurement (i.e. week 1) of the control group to illustrate the changes caused by different conditions. In the (PONT) group, as compared with the control group, there was a significant reduction of P1 and PhNR amplitudes in all retinal regions a week after surgery (p<0.01), while the N1 amplitude was not affected by the PONT procedure (Figure 4).

### Effect of PBS with PONT

**Figure 5:**
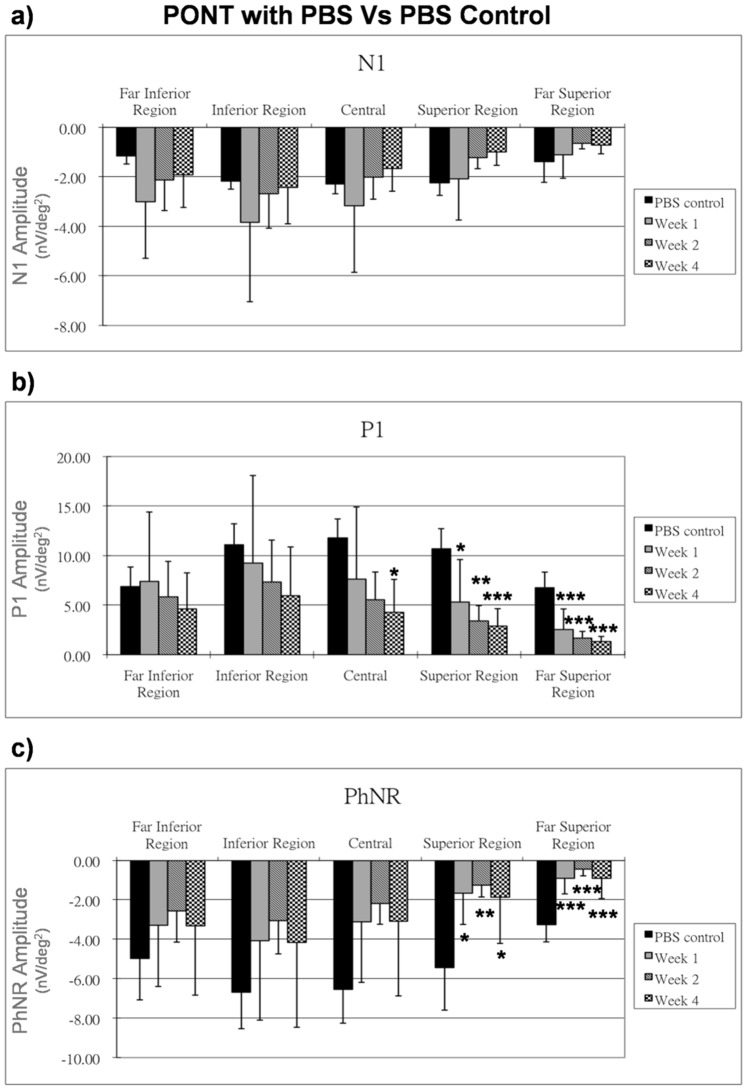
Effect of PBS on mfERG in PONT group. The effect of PBS on the mfERG responses (nV/deg^2^) at one, two and four weeks after PONT are shown. The responses are compared with those of the PBS control group. Bars = 1 SD; *p<0.05; **p<0.01; ***p<0.001.

Feeding with PBS a week prior to PONT, the N1 amplitude from the (PONT+PBS) group was not affected (Figure 5a) but the P1 amplitude showed a significant reduction in the superior regions after PONT for all three time points (p<0.05); it was also significantly reduced in the central region 4 weeks after PONT (p<0.05), whereas the P1 amplitude showed only a gradual reduction in the inferior retina when compared with the PBS control group (Figure 5b). The PhNR amplitude showed a significant reduction in the superior retina after PONT for all three time points (p<0.05), but its amplitude was not significantly reduced in the inferior retina for any time points (Figure 5c).

### Effect of LBP with PONT

**Figure 6:**
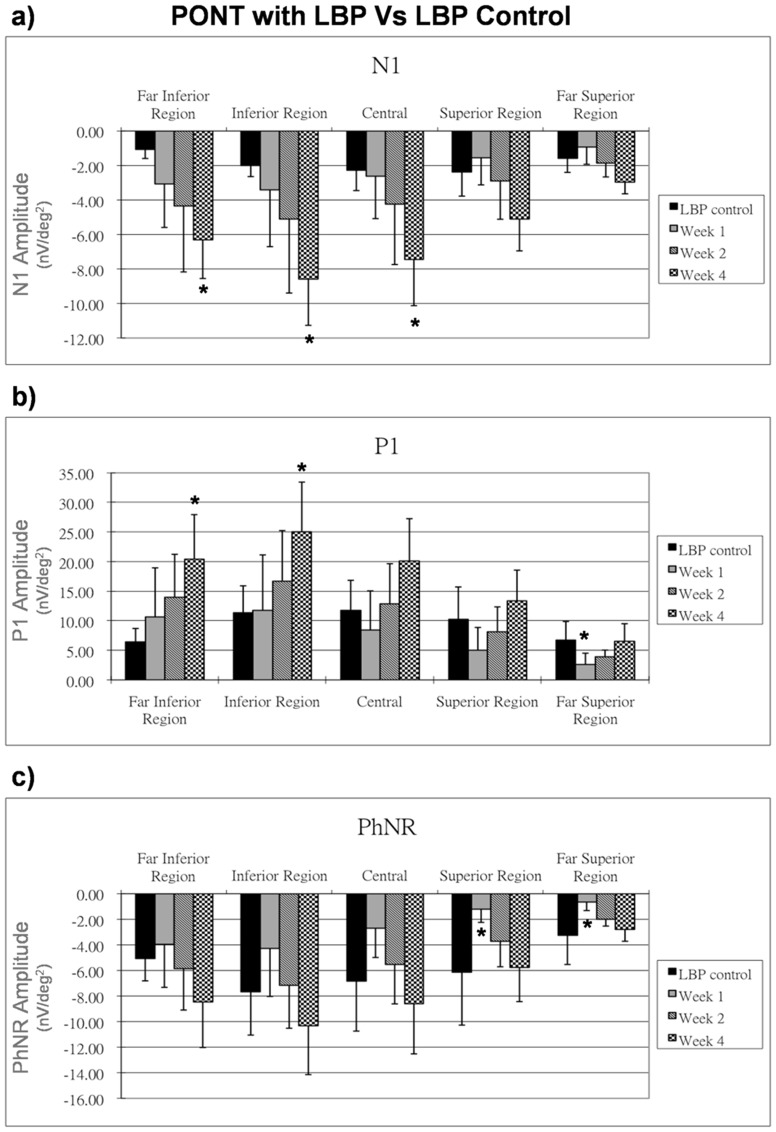
Effect of LBP on mfERG in PONT group. The effect of LBP on the mfERG responses (nV/deg^2^) at one, two and four weeks after PONT are shown. The responses are compared with those of the LBP control group. Bars = 1 SD; *p<0.05; **p<0.01; ***p<0.001.

After feeding with LBP a week prior to PONT, the (PONT+LBP) group showed increased N1 responses, P1 responses and PhNR responses, especially in the inferior retina as compared to the LBP control group. The N1 amplitudes were significantly increased at week 4 after PONT (p<0.05) except in the superior regions (Figure 6a). The P1 amplitude in the far superior region showed a significantly reduction 1 week after PONT (p<0.05) but then returned to the normal range. P1 amplitudes remained normal in other regions after PONT but were significantly increased in the inferior retina 4 weeks after PONT (p<0.05) (Figure 6b). The PhNR amplitude reduced significantly in the superior retina 1 week after PONT and then gradually returned to the normal range. The PhNR amplitude in the inferior retina appeared to be increased after PONT with prolonged feeding with LBP, but this effect was not statistically significant (Figure 6c).

### Comparison of the Effects of LBP and PBS

**Figure 7:**
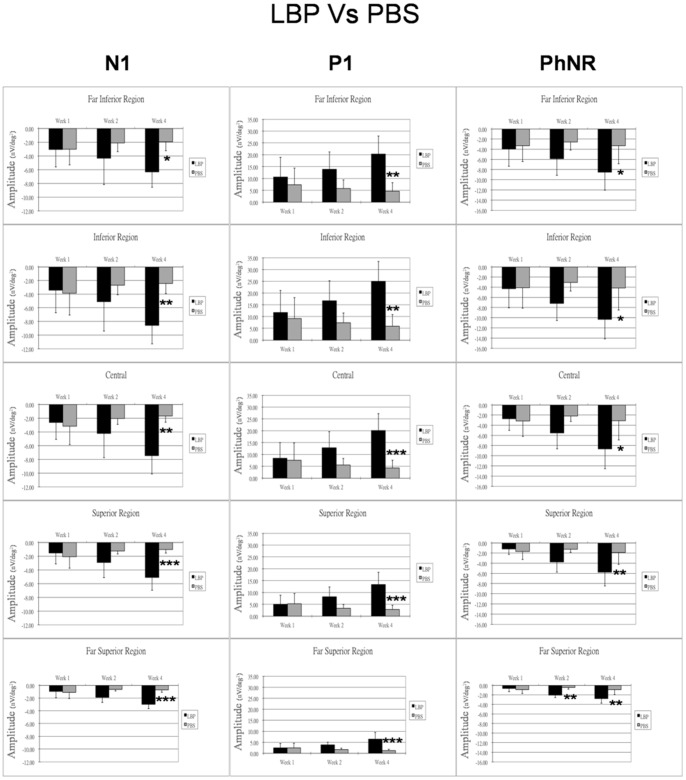
Comparison of the effects of LBP and PBS on mfERG. The effects of LBP and PBS on the mfERG responses (nV/deg^2^) after PONT for different retinal regions are shown. Bars = 1 SD; *p<0.05; **p<0.01; ***p<0.001.

**Figure 8:**
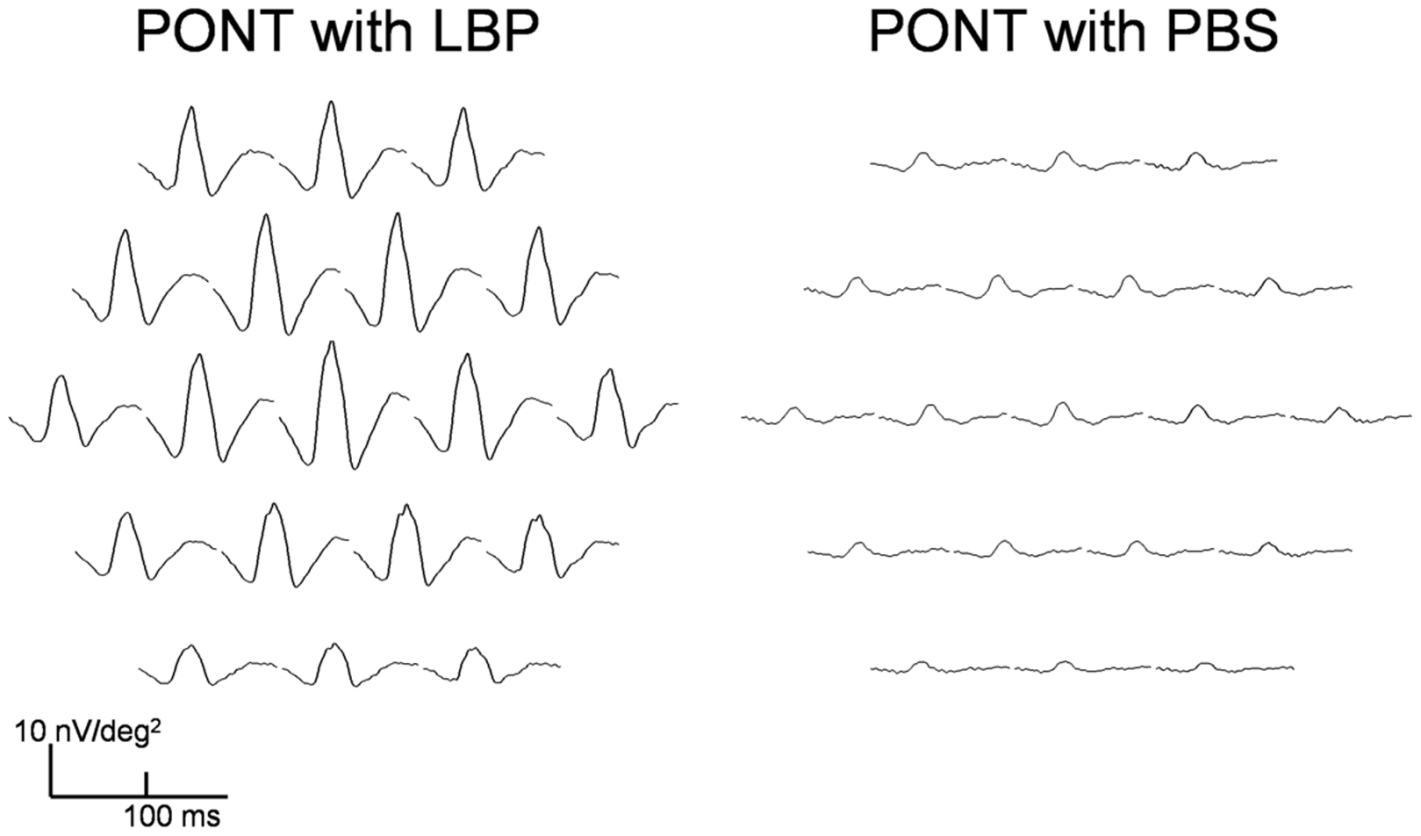
Multifocal ERG traces array with LBP and PBS. The response traces from the right eye of a SD rat in the PONT+LBP group (right) and a SD rat in the PONT+PBS group (left) 4 weeks after PONT.

The comparison between the (PONT+LBP) group and the (PONT+PBS) group showed that the amplitudes of all mfERG components are comparable at all retinal regions one week after PONT. Since the retinal function in the PBS group was reducing and the retinal function in the LBP group was increasing, there were observable differences of the amplitudes between groups two weeks after PONT. Significantly larger amplitudes of all mfERG components were found in the LBP group for all regions four weeks after PONT (p<0.05) (Figure 7). The mfERG waveforms from the LBP and the PBS groups 4 weeks after PONT are shown in Figure 8.

### Effect of LBP, PBS, TTX and NMDA

**Figure 9:**
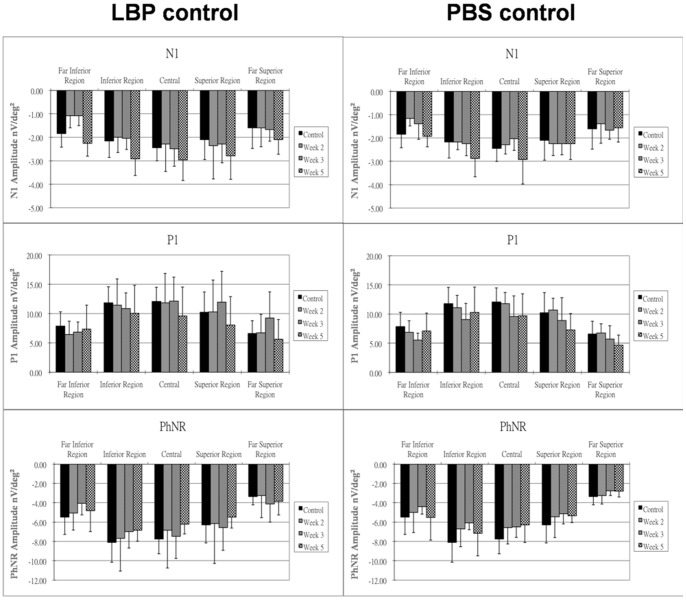
Effect of LBP or PBS on mfERG. The changes of mfERG responses (nV/deg^2^) after prolonged feeding with LBP or PBS for two, three and five weeks are shown. Bars = 1 SD; *p<0.05; **p<0.01; ***p<0.001.

In the groups fed with LBP (LBP group) or PBS (PBS group) without PONT, there were no significant differences in amplitude for any mfERG components among all retinal regions (Figure 9). The drugs applied in the control animals at the end of the experiment (TTX and NMDA) were to investigate the retinal origins of the components of the rat mfERG. After application of TTX alone or together with NMDA, there were no remarkable changes in the amplitude of N1 or P1 at the visual streak. However, the amplitude of PhNR showed a significant reduction after the administration of TTX (p<0.01), and its amplitude further reduced after additional application of NMDA (p<0.001) (Table 2).

**Table 2:** Effect of drug administration on mfERG.

mfERG response amplitude (nV/deg^2^)			
	N1	P1	PhNR
**Before drug administration**	−4.32±0.68	10.04±2.45	−4.20±1.04
**TTX**	−3.72±0.90	9.50±2.41	−3.28±1.16*
**NMDA**	−3.72±0.72	9.68±2.69	−2.92±1.34**

The mfERG amplitudes of different components along the visual streak before and after drug administration in control group are listed. (TTX – Tetrodotoxin; NMDA – N-methyl-D-asparatic acid; PhNR – photopic negative response) (*p<0.01; **p<0.001).

## Discussion

Retinal functional changes caused by PONT and the effect of LBP in preserving visual function after primary injury and secondary degeneration have been demonstrated in this study.

### Origins of SD Rat mfERG

Our results illustrate that the slow-stimulation mfERG paradigm produced topographical retinal responses for the rat. Unlike primates, the optic nerve head is located approximately at the center of the rat retina, and the rat retina lacks a defined macular region. The majority of cone cells as well as ganglion cells are localized in a band in the central retina forming the visual streak; the most dense cone region is located slightly temporal to the optic nerve head. [32] The topographical distribution of the cone pathway is mirrored by the greater mfERG response in the central field with a peak in the nasal field.

In the mammalian mfERG, the main contribution to the first-order response is from the outer retina, where the N1 involves responses from cone photoreceptors and OFF-bipolar cells; the leading edge of P1 is dominated by ON-bipolar cell activity. [18], [19] Our study further demonstrates the retinal origins of the rat slow-stimulation mfERG by using an established pharmacological suppression method to inhibit the inner retinal contributions. TTX inhibits the voltage-gated sodium channel in the ganglion cells and some amacrine cells, and is powerful in removing any partial inner retinal contribution to the mfERG. [31] NMDA is an ionotropic glutamate agonist which removes the remaining inner retinal activity that is not suppressed by TTX. [26] After the administration of TTX+NMDA, the insignificant changes of the N1 and P1 amplitudes suggests that these rat mfERG components were generated mainly from activity of the outer retina. Moreover, the stimulus frequency of the slow-stimulation mfERG paradigm used in this study was around 6 Hz, which can evoke the mfERG PhNR. [17] Our findings showed that the mfERG PhNR amplitude was diminished after TTX and/or NMDA administration; this suggests that this component is mainly generated from the inner retina (retinal ganglion cells and amacrine cells). This is consonant with the notion that the PhNR from the conventional flash ERG in rodents originates predominately from the activity of amacrine cells. [17] However, the effect of TTX may be weakened by the isoflurane that was used for anesthesia, since isoflurane can suppress retinal ganglion cell activity. [18], [33] Isoflurane will only suppress the components associated with the retinal ganglion cells activity by about 23%, and TTX produces a further 33% suppression of these components. [26] In addition, after the administration of TTX+NMDA, the amplitude of the PhNR was only reduced by 30%. This implies that the PhNR from the slow-stimulation mfERG paradigm may not solely be generated by the inner retinal activities as it is in the flash ERG.

### The Effect of PONT on Retinal Function

Histological studies have shown that degeneration of inner retinal cells occurs after optic nerve transection [34] and PONT. [11] A substantial loss of retinal ganglion cells and their axons in the inferior retina was found when the dorsal part of optic nerve had only been transected in Wistar rats and PVG Hooded rats [10], [13], [14], although the loss was not as much as that in the superior retina where the primary injury occurred. [9], [10] It was assumed that the partial transection of the dorsal optic nerve led only to a primary and direct injury in the superior part, but not in the inferior part, of the optic nerve. [35] Loss of ganglion cells in the inferior retina was assumed to be caused by secondary degeneration; [9], [13] it has been speculated that secondary degeneration may cause ganglion cell loss as an indirect effect of the death of the directly injured ganglion cells [9], [10], [13].

The functional assessment in this study showed that the secondary degeneration caused by PONT was not limited to the affected retinal layer, but deterioration of outer retinal function was also found. After PONT, the reduced PhNR in the inferior retina indicated malfunction of inner retinal activity in that region. Some anatomical findings have shown a significant difference in ganglion cell death between the superior and inferior retina a week after partial optic nerve transection. [9] Although a non-significant difference was found in our previous study, [11] the comparable reduction of the PhNR between the superior and the inferior retina suggests that functional changes of the inner retinal activities may occur prior to the cell apoptosis in optic neuropathy. [36] Further study by performing serial mfERG testing for a few days after PONT, where the death of retinal ganglion cells is not obvious, would be of interest. In addition, the P1 amplitude was decreased in the whole retina. Since the P1 component is attributed mainly to activity of ON-bipolar cells, [18], [19] the functional alteration caused by the PONT indicates that the influence of secondary degeneration is not solely limited to the adjacent cells in horizontal layers, but it may also extend vertically through the retina, from inner to outer retinal layers. Although most studies have only focused on the histological changes in inner retinal cells after PONT, [10], [13] the degeneration of amacrine cells after optic transection [34] supports our speculation that secondary degeneration can adversely affect retinal layers beyond retinal ganglion cell level.

### The Effect of LBP on Retinal Function after PONT

LBP has a neuroprotective effect by reducing the loss of retinal ganglion cells in ocular hypertension [4], [5], [6] and PONT. [11] Around 70% of ganglion cell death in ocular hypertensive rats can be retarded with a short-term feeding of LBP and this neuroprotective effect can be maintained for up to 4 weeks. [4] It is believed that the neuroprotective effect of LBP is partly due to modulating the activation of microglia, [5] as manipulating the activation state of microglia is beneficial for neuron protection. [37] It has also been suggested that the survival of ganglion cells may be mediated by an increase in expression of *β*B2 crystallin [6] which is a neuroprotective agent. LBP can also decrease secondary degeneration of retinal ganglion cells (RGCs) after PONT via inhibiting oxidative stress and activation of c-jun N-terminal kinase (JNK) pathway [11].

In this study, we demonstrated that the LBP has an effect on altering abnormal retinal function caused by PONT. A week after PONT, the response amplitudes of the mfERG components including the outer retinal responses in either LBP or PBS groups were reduced similarly in the superior retina; this suggested that the effect of degeneration outweighed the effect of the LBP in the early stage, or a week of LBP feeding may not be long enough to provide a strong effect for such an initial traumatic insult. Under prolonged LBP feeding, the retinal function increased in both superior and inferior retina, and the mfERG amplitudes were significantly larger than those in the PBS group 4 weeks after the PONT. This suggests that LBP influenced both the inner and outer retinal response. Although both the outer and inner retinal responses were found to be enhanced in the LBP group after PONT, it is still not clear how much of this enhancement of inner retinal function reflects an expression of photoreceptor or bipolar cell response enhancement and how much of this is regulated solely by any neuroprotective effect on the inner retina. [11] Considering the findings of previous histological studies [4], [6] and this functional study, the effect of LBP seems most likely act on both outer and inner retina.

In the LBP group with PONT, the PhNR in the superior retina initially reduced and then recovered their response to normal levels. Since the retinal ganglion cells in this particular region suffered more from direct injury due to PONT than the inferior retina, and the number of ganglion cells has been shown to be significantly reduced 4 weeks after PONT, [10], [11] the normal PhNR in this study indicates that this component may not simply reflect ganglion cell function but is more likely to be generated from amacrine cells [34] with partial contribution from the outer retinal response. Therefore, it is still difficult to know how the inner retinal function was preserved by the LBP.

Nevertheless, the degeneration caused by PONT is not limited to the inner retina, and LBP could alter the abnormal retinal function caused by the PONT. Interestingly, the use of PBS seems to reduce the effect of PONT on retinal function. Although further investigation is needed for this finding, the significantly larger amplitudes of all mfERG components found in the LBP+PONT group than those in the PBS+PONT group for all regions four weeks after PONT imply that LBP could reduce the functional deterioration caused by PONT.

Furthermore, although all the parameters of the mfERG across the whole retina were preserved after PONT, the effect of LBP on retinal function was different between the superior and inferior retina. With the treatment of LBP, the retinal responses had returned to normal in the superior retina 4 weeks after PONT; however, some super-normal responses (nearly twice normal) were noticed in the inferior retina where the secondary degeneration is likely to have occurred. This phenomenon, however, did not occur in those eyes without PONT either under prolonged feeding of LBP or PBS feeding, where all the responses remained nearly constant. If LBP enhances response, super-normal mfERG responses would also be expected in the LBP control group. Therefore, the super-normal mfERG responses with LBP in the PONT group should not be simply due to preventing degeneration or health improvement. LBP alone did not increase the mfERG response, or upregulate the expression of *β*B2 crystallin. [6]. Hence, we believe that the effect of the LBP is activated only in the presence of PONT. Since the implicit times of the mfERG were not changed, the super-normal mfERG responses may be caused by changing the conductivity of the photoreceptors, electrical resistance of the neurons, improving the synaptic transmission as well as altering blood flow to the retinal tissues that enhances the electro-retinal activity. Further histological study on the outer retinal cells after PONT should provide evidence to help assess this speculation.

Communication between the inferior retina and the cortex was still intact after PONT. [35] There was still some ganglion cell loss in the inferior retina after PONT although the rate of apoptosis was retarded by prolonged feeding with LBP. [11] Signal transmission was interrupted to a degree, and the super-normal mfERG responses with LBP under PONT may be a compensation reflex to compensate for the signal disturbance at the inner retinal level, by enhancing the signal generated from the outer retina. In this study, LBP reduced the deterioration of retinal function after PONT and the secondary degeneration due to PONT was not only limited to the ganglion cell layer, but appeared to be a widespread effect also affecting the outer retinal layer(s).
